# Comment on Kura et al. Cost-Effectiveness Analysis of Clesrovimab for Respiratory Syncytial Virus in Infants in the United States. *Vaccines* 2026, *14*, 411

**DOI:** 10.3390/vaccines14090758

**Published:** 2026-08-31

**Authors:** Erin N. Hodges, Mehdi Ghemmouri, Maureen P. Neary, Veronica Gabriel, Cecilia Ottaviano, Karine Mari, Veronica Ottobrino, Ayman Chit, Joseph Domachowske, Benjamin Yarnoff, Robert Musci, Leonard R. Krilov

**Affiliations:** 1 Global Medical Affairs, Sanofi Vaccines US, Inc., Morristown, NJ 07960, USA; 2 Global Health Economics and Value Assessment, Sanofi Vaccines, 69007 Lyon, France; mehdi.ghemmouri@sanofi.com (M.G.); veronica.ottobrino@sanofi.com (V.O.); 3 North America Health Economics and Value Access, Sanofi Vaccines US, Inc., Morristown, NJ 07960, USA; maureen.neary@sanofi.com (M.P.N.); veronica.gabriel@sanofi.com (V.G.); 4 North America Medical Affairs, Sanofi Vaccines US, Inc., Morristown, NJ 07960, USA; cecilia.ottaviano@sanofi.com (C.O.); ayman.chit@sanofi.com (A.C.); joseph.domachowske@sanofi.com (J.D.); 5 Biostatistics, Sanofi Vaccines, 69007 Lyon, France; karine.mari@sanofi.com; 6 Leslie Dan Faculty of Pharmacy, University of Toronto, Toronto, ON M5S 3M2, Canada; 7 Health Economics and Market Access, Evidera Inc., Wilmington, NC 28401, USA; benjamin.yarnoff@thermofisher.com (B.Y.); robert.musci@thermofisher.com (R.M.); 8 Pediatric Infectious Disease Researcher, Woodbury, NY 11797, USA

**Keywords:** respiratory syncytial virus, RSV, clesrovimab, nirsevimab, cost-effectiveness, modeling

We read with interest the study by Kura et al. [1] evaluating the potential clinical and economic impact of clesrovimab compared with nirsevimab, palivizumab, and maternal RSVpreF vaccination for the prevention of respiratory syncytial virus (RSV) lower respiratory tract infections (LRTIs) in infants in the United States. The authors’ conclusion that clesrovimab is cost-saving versus nirsevimab relies on a number of assumptions in the base case that appear to be biased to favor clesrovimab, including a post hoc efficacy endpoint constructed for clesrovimab and duration of protection (i.e., durability of efficacy) that is inconsistent with the FDA label for clesrovimab. We found that the publication also does not include scenarios using alternative efficacy estimates for clesrovimab that would help in understanding the results of the model. Additional transparency and the inclusion of scenario analyses would support a more robust interpretation by immunization program decision-makers, payers, and health systems.

The authors use a post hoc efficacy endpoint as the input for protection with clesrovimab. By constructing an endpoint after the trial was unblinded, the authors assume an efficacy of 87.2% (95% CI: 75.1–93.4%) through 180 days against medically attended RSV-associated lower respiratory tract infection (MA-LRTI), rather than relying on the primary prespecified endpoint of the study, with a point estimate of 60.4% (95% CI: 44.1–71.9%) through 150 days [2], with non-overlapping confidence intervals, suggesting a possible lack of consistency of the newly constructed post hoc efficacy endpoint with the primary efficacy endpoint. However, since these estimates arise from different endpoint definitions within the same clinical trial population, formal statistical comparisons would require methods that account for the dependence between estimates.

The authors state that the post hoc analysis was an attempt to harmonize the clesrovimab endpoint with that used in nirsevimab trials to enable cross-trial comparison. There is no statement in the paper addressing specifically what steps were taken for this harmonization, regarding expert external clinical panel inputs, testing in alternative databases, etc. However, when we conducted an expert clinical review, we observed meaningful discrepancies in the definition and assessment of RSV-associated MA-LRTI for the different primary endpoints used in the clesrovimab and nirsevimab trials (Table 1) [2,3,4,5,6], which may reflect differences in severity criteria and data collection approaches across the two clinical programs.

In addition, the use of this post hoc assessment as the input in a comparative model appears to disregard several important considerations. First, over 60% of RSV MA-LRTI cases that met the primary case definition were excluded using the post hoc endpoint definition, and exclusions were disproportionately concentrated in the clesrovimab arm. This yielded only 10 cases meeting the post hoc endpoint definition in the clesrovimab arm, greatly increasing sensitivity to individual case variations, with broad confidence intervals [2]. Second, differences in the actual signs and symptoms collected across the nirsevimab and clesrovimab clinical trials reflect variations in study design, including differences in severity criteria used and likely in specific data collected in case report forms. Third, this analysis was not prespecified. With no prespecified alpha adjustment or gatekeeping, the risk of Type I error inflation is substantial, and any nominally significant findings cannot be interpreted as confirmatory evidence.

Consequently, this approach does not robustly address the goal of endpoint alignment but rather introduces concerning limitations regarding the interpretation of model results. Post hoc analyses serve a valuable role as exploratory tools generating hypotheses for future research, but their evidentiary weight should reflect their exploratory nature.

Indeed, Zar et al. themselves characterized this analysis as exploratory, intended to facilitate indirect comparisons across RSV trials, and explicitly noted the limitations of cross-trial comparisons arising from differences in study populations, surveillance methods, and endpoint definitions [2]. This exploratory framing by the trial authors further supports our primary concern: an endpoint acknowledged as hypothesis-generating should not have been used as the principal efficacy input in a base-case cost-effectiveness model. The prespecified primary endpoint should have served as the base case, with the post hoc estimate explored, if at all, only as a scenario analysis, with its stated limitations explicitly acknowledged.

Using a post hoc analysis as the primary efficacy endpoint in a cost-effectiveness model’s base case while applying a primary endpoint to the comparator conflates exploratory and confirmatory evidence. Also, the FDA guidance states that exploratory endpoints are generally not used to support conclusions [9]. Overall, the use of this newly constructed post hoc efficacy endpoint creates some asymmetric evidence that appears to directionally favor clesrovimab in the cost-effectiveness analysis.

The lack of scenario testing using alternative efficacy estimates for clesrovimab to assess the robustness of conclusions using this key assumption further compounds these endpoint limitations. This gap is distinct from parameter uncertainty: the deterministic sensitivity analysis presented by the authors characterizes uncertainty around the selected post hoc efficacy estimate but does not address the basic, structural question of whether that estimate, rather than the prespecified primary endpoint, is the appropriate base-case input. The model’s own analysis identifies clesrovimab efficacy as one of the variables driving cost-effectiveness conclusions, yet this critical dependency on a post hoc endpoint, together with the aforementioned concerns about this endpoint, is not discussed in the study’s limitations section. Notably, this approach is not consistent with established guidance for modeling.

ISPOR-SMDM Good Research Practices [10] require that “populating models with parameter estimates should conform to evidence-based medicine principles” and that “for DSA, an interval estimate representing beliefs about the parameter’s plausible range is required.” NICE Health Technology Evaluations guidance [11] further states that “when more than 1 plausible set of [input] data is available, sensitivity analyses should be done to show the effect of the alternative” values. The HAS Methodological Guide for Economic Evaluation [12] specifies that “in the absence of a solid argument clearly justifying one methodological choice over other credible alternatives, it is recommended to retain the option least favorable to the evaluated intervention in terms of cost or health outcome differential”, a principle directly applicable here, where the post hoc estimate (87.2%) yields substantially more favorable conclusions than the prespecified primary endpoint (60.4%).

Beyond the limitations for post hoc efficacy endpoint, the model assigns 6 months of protection to clesrovimab and 5 months to nirsevimab, justified solely by the maximum follow-up time in the respective registrational trials. This assumption for clesrovimab is inconsistent with FDA Prescribing Information, which specifies 5 months of protection [13]. Additionally, the estimate for duration of efficacy (durability of efficacy) over the period from day 150 to day 180 is based on observations of only one event each in the clesrovimab and placebo groups during that one-month time period. Specifically, as shown in Figure 1 of the NEJM publication, cases in the clesrovimab group increased from 10 at day 150 to 11 at day 180, and from 41 to 42 in the placebo group [2]. Furthermore, the same HAS principle cited above applies here, favoring scenarios assuming equivalent protection windows for both nirsevimab and clesrovimab (e.g., 5 months each or 6 months each), consistent with regulatory assessments.

The authors rely solely on the phase 3 trial follow-up periods to justify this selection, while failing to acknowledge the phase 3b HARMONIE study for nirsevimab that demonstrated sustained efficacy of 82.7% (95% CI: 67.8–91.5%) against RSV hospitalization through 180 days after dosing [14].

Moreover, pharmacokinetic analyses show nirsevimab serum persistence and elevated neutralizing antibody levels through 360 days [15,16], and real-world evidence from the United States, Spain, and Chile supports nirsevimab effectiveness through the entire RSV season and up to 175 days after administration [17,18,19,20]. Additionally, FDA Prescribing Information reports a half-life of 71 days for nirsevimab and 44 days for clesrovimab in infants [13,21,22,23]. Given this shorter half-life and that the majority (>80%) of RSV cases in the CLEVER efficacy trial occurred within the first 100 days after dosing [24], we previously conducted pharmacokinetic modeling using comparative antibody serum concentration analyses [7]. This analysis estimated that clesrovimab reaches comparable antibody levels at day 96 to those demonstrated by nirsevimab at day 150, informing a lower-bound scenario of 100 days for duration of cumulative efficacy. While the connection between antibody serum concentration and clinical duration of protection is suggestive rather than conclusively established, in the absence of a formal correlate of protection for RSV, this pharmacokinetic evidence further supports that the duration of protection for clesrovimab is not likely to be longer than that of nirsevimab.

The methods and scientific rationale for these input selections were clearly explained in support of our published model. We also evaluated a 150-day scenario aligned with the FDA label to account for uncertainty in the duration of cumulative efficacy.

In contrast, the base-case scenario in the Kura et al. publication fails to provide scientific justification. We acknowledge that Kura et al. did include a scenario analysis assuming equal five-month protection for both products (Scenario 1.2). However, this equal-duration assumption was not adopted as the base case, and instead, a six-versus-five-month differential was used as the primary analysis, which is unsupported by regulatory and real-world evidence.

The authors further characterize our previous analyses (Yarnoff et al. [7] and Kieffer et al. [25]) as introducing structural bias by mixing real-world effectiveness estimates for nirsevimab with trial-based efficacy for clesrovimab. However, the Yarnoff et al. analysis explicitly addressed this concern by also conducting a scenario analysis using randomized trial-based efficacy for both products simultaneously. For preterm infants (<35 weeks gestational age), efficacy was 86.5% (inpatient) and 86.2% (outpatient) for nirsevimab versus 86.8% (inpatient) and 79.8% (outpatient) for clesrovimab; for late preterm and term infants (≥35 weeks gestational age), efficacy was 76.8% (inpatient) and 76.4% (outpatient) for nirsevimab versus 82.5% (inpatient) and 52.6% (outpatient) for clesrovimab [2,4,6]. Notably, in this analysis, nirsevimab remained dominant across all tested duration of protection scenarios, preventing 40–82% more RSV cases and incurring 7–12% lower total costs. Additionally, the claim that the Kura et al. analysis provides a more accurate evaluation by consistently applying trial-based efficacy is undermined by its reliance on a post hoc, unblinded endpoint for clesrovimab rather than the prespecified primary endpoint used in the registration trial.

In addition, Kura et al. cite the University of Michigan–CDC modeling as supporting their conclusions. However, the UM-CDC analysis modeled clesrovimab and nirsevimab separately using different assumptions (including different antibody waning patterns and outcome severity criteria informing the efficacy inputs), which introduces differences that prevent a direct comparison of the results [26,27]. While a range of modeling approaches can be informative, citing this work as validation does not remove the fundamental flaws introduced by the post hoc endpoint construction/selection and duration assumptions raised above.

The assumptions adopted in our previously published analyses represent one set of scientifically plausible modeling choices among several alternatives. Rather than advocating for a single correct approach, we emphasize the need for comprehensive sensitivity analyses exploring alternative plausible assumptions in the Kura et al. study. We note that all currently available comparisons between clesrovimab and nirsevimab remain indirect, and that differences in study populations, trial conduct, RSV epidemiology, outcome ascertainment, and healthcare systems introduce additional uncertainty that cannot be fully eliminated through endpoint harmonization alone.

In summary, the methodological concerns raised throughout the Kura et al. analysis appear to collectively bias the model conclusions in favor of clesrovimab, limiting the interpretability of comparative effectiveness findings. The analyses should incorporate transparent assumptions, a clearly documented rationale for key modeling choices, sensitivity testing across alternative efficacy inputs, particularly using prespecified clesrovimab endpoints, explicit discussion of the limitations inherent in cross-trial comparisons, and post hoc endpoint harmonization. This type of detail is needed to provide healthcare decision-makers with a complete and robust characterization of the evidence base. We welcome the authors’ response to these methodological points.

## Figures and Tables

**Table 1:** RSV case definition comparisons: nirsevimab and clesrovimab trials [7].

	Clesrovimab Efficacy Trial Primary Endpoint (RSV MALRI1) (NCT04767373)	Clesrovimab Efficacy Trial Post Hoc Endpoint (RSV MALRI2) (NCT04767373)	Nirsevimab Efficacy Trials Primary Endpoint (MA RSV LRTI) (NCT02878330 & NCT03979313)
RSV-positive RT-PCR Test	Yes, required	Yes, required	Yes, required
Lower Respiratory	N/A	At least one of: Rhonchi Rales/Crackles Wheeze	At least one sign on examination: Rhonchi Rales Crackles Wheeze
Sign/Symptom	Cough or Difficulty Breathing required	N/A	N/A
Severity	At least one of: Tachypnea ^a^ (increased respiratory rate) Hypoxemia ^b^ Chest wall in-drawing/retraction Dehydration due to respiratory symptoms Wheezing Rales/Crackles	At least one of: Tachypnea (increased respiratory rate) Hypoxemia Chest wall in-drawing/retraction Dehydration due to respiratory symptoms	At least one of: Increased respiratory rate ^c^ Hypoxemia ^d^ Acute hypoxic or ventilatory failure New onset apnea Nasal flaring Retractions Grunting Dehydration due to respiratory distress

Abbreviations: CHD = congenital heart disease; CLD = chronic lung disease; LRI = lower respiratory infection; LRTI = lower respiratory tract infection; MA = medically attended; N/A = not applicable; RSV = respiratory syncytial virus; RT-PCR = reverse transcriptase polymerase chain reaction; Sp02 = oxygen saturation as measured by pulse oximetry; RSV MALRI1: RSV-associated MALRI with ≥1 indicator of LRI or disease severity [2]; RSV MALRI2: RSV-associated MALRI with ≥2 indicator of LRI or disease severity [8]; MA RSV LRTI: MA RSV-associated LRTI [3,4]; ^a^ respiratory rate ≥60 breaths per minute for <2 months of age; ≥50 breaths per minute for 2 to 12 months of age; or ≥40 breaths per minute for 13 to 24 months of age. ^b^ Sp02 <95% on room air at sea level, <92% on room air at altitude >1800 m, or 5% or more below baseline level in children with CLD or CHD with chronic underlying hypoxemia. ^c^ age: <2 months, ≥60 breaths/min; 2 to 6 months, ≥50 breaths/min; >6 months, ≥40 breaths/min. ^d^ in room air: oxygen saturation <95% at altitudes ≤1800 m or <92% at altitudes >1800 m.

## Data Availability

No new data were created or analyzed in this study. Data sharing is not applicable to this article.
